# Range Expansion of Obligate Antagonists but Not Mutualists in a Desert Fig (*Ficus petiolaris*)

**DOI:** 10.3390/plants15071012

**Published:** 2026-03-26

**Authors:** Molly Gans, Judith L. Bronstein

**Affiliations:** Department of Ecology and Evolutionary Biology, University of Arizona, Tucson, AZ 85721, USA; judieb@arizona.edu

**Keywords:** mutualism, cheating, obligate associations, fig-fig wasp interactions, range shifts

## Abstract

Species may respond to climate change by shifting habitats. For species that engage in obligate interactions, however, such range shifts are only possible if the interaction can occur in the extended range, either via dispersal of the original interaction partner or by acquisition of a novel interaction partner in the extended range. To explore how obligate interactions may persist by parallel dispersal of partners, we investigated the community of obligate fig wasps associated with an isolated fig tree (*Ficus petiolaris*) in Arizona, USA, found 200 km north of its native range. We expected that if the fig wasps (the single obligate pollinator as well, perhaps, as species-specific non-pollinating wasp associates) were occasionally acquired via dispersal from the native range, then (a) there would be months in which fig wasps were absent, and (b) fig wasps would not be genetically differentiated from those in the native range. Conversely, if fig wasps had formed persistent populations in Arizona on the single tree, we expected that (a) the tree would exhibit high reproductive asynchrony, a trait necessary for long-term pollinator persistence, and (b) the pollinator species would be present throughout the study. We collected and identified fig wasps from this tree for 20 months to determine the composition of the community as well as presence/absence patterns. We also used existing genetic data from two species of non-pollinating fig wasps from Arizona to determine their geographic origin and the extent of genetic differentiation from the same species in the native range. We found a persistent fig wasp community, but one consisting almost exclusively of at least two non-pollinating species in the genus *Idarnes*; the pollinator species was virtually absent. Our results suggest that previously unknown aspects of the natural history of *Idarnes* have allowed it to follow its fig tree host out of their shared range. These results point to several puzzles about range extension, as well as a testable hypothesis that the two *Idarnes* species groups can only persist without pollinators when they occur in tandem. Future ecological and genomic studies are needed to resolve these issues.

## 1. Introduction

Global climate change is altering where species live. One way this occurs is through range shifts, as species move poleward or upslope into formerly unsuitable areas while at the same time experiencing local extinctions in formerly suitable areas that are no longer habitable [1,2]. Biotic interactions also play an important role in determining the geographic ranges of species and how they may shift under climate change [3,4,5,6,7,8]. Obligate associations are likely to respond more strongly to disruption than facultative associations [9]. Obligate associations can persist beyond their range, and thus potentially extend that range, by at least two mechanisms: either obligate associates can follow their partners into new locations, or species can acquire partners native to the new location that can functionally replace those associates that were left behind [10]. Whether either of these is possible depends on the nature of the interaction (e.g., how easily replaced a function is) [11].

Fig trees and their obligate, highly specialized fig wasp associates serve as a test case for how interactions are affected as species move beyond their current ranges. Figs (*Ficus*, Moraceae) are pollinated by wasps in the family Agaonidae (Chalcidoidea), which reproduce within fig inflorescences, called syconia [12]. Fig species are either monoecious or gynodioecious [12]. In the monoecious species, female pollinator wasps (termed foundresses) enter a receptive syconium through a small bract-lined pore (the ostiole), pollinate female flowers, and lay eggs in some ovules [12]. Foundresses die, almost always trapped inside, although female pollinators are known to move between syconia on a tree [13]. Once the seeds and wasps have matured, males of the pollinating species emerge into the syconium cavity and mate with their sisters, often still enclosed within ovules. After mating, female pollinator wasps collect pollen from male flowers and exit syconia through a hole chewed by males of the pollinator species.

The highly synchronized life histories of figs and fig wasps would appear to make range shifts particularly difficult. Pollinator females are minute in size (<2 mm), only live for a few days, and can only oviposit into syconia during a narrow window of time of syconia receptivity (a brief interval marked by release of a species-specific volatile signal undetectable to the human nose) [14,15,16]. Individuals of most fig species are relatively tightly synchronized with regard to phenological stage, but out of synchrony with other fig individuals within the same population [17,18], forcing pollen-laden fig wasps to depart their natal tree, leading to outcrossing. Without nearby trees in the receptive phase co-occurring with individuals from which mature pollinator females are escaping, the relationship is doomed. The isolation of fig trees, either due to loss of individuals within a population or to individuals being planted or dispersing naturally well beyond their natural range, makes reproduction of either partner unlikely because pollinators will not survive long enough to successfully locate a syconium in the correct and brief phase.

Surprisingly, however, pollinating fig wasps are able to locate isolated trees, on occasion dispersing thousands of kilometers [19,20,21]. Because many fig species have been introduced to new regions as ornamentals, there have been ample opportunities to observe this occurrence, particularly when it is not a single event but rather results in the establishment and growth of a local fig population. Some introduced figs have become invasive upon the arrival and subsequent establishment of their pollinators (e.g., *Ficus microcarpa*, an Asian species, in Europe and the Neotropics [22,23,24]). In contrast, other fig species introduced far beyond their native range have until recently remained without an associated pollinator population (e.g., *F. elastica* in Singapore, [25]). Fig species differences in persistence beyond the range may, at least in part, be a function of how suitable the conditions of the new range are for larval development of the fig wasps as well as the adult, free-flying stage [26]. In many cases, it is also necessary for there to have been buildup of a sufficiently large fig tree population to support pollinators [27]. It has been conjectured that in some species, syconia on a given individual tree might always be sufficiently out of synchrony for the pollinator population to cycle indefinitely on a single tree [28], but evidence is lacking [12].

Numerous other wasp species from various families of Chalcidoidea also obligately reproduce within syconia but do not pollinate [29,30]. These are commonly termed non-pollinating fig wasps (hereafter, NPFWs) [31]. NPFWs exhibit a wide range of life histories; all are minute in size, but exhibit exceptionally diverse feeding niches [31]. What unites them ecologically is their failure to collect, carry, and deposit pollen. NPFW females lay their eggs, mostly directly into the ovules, either from outside by ovipositing through the walls of syconia or by entering syconia [31]. After the seeds and wasps have developed, males of some NPFW species mate with females while still inside the syconium cavity, similar to pollinator species, whereas other NPFW species mate on the surface of their natal syconium [31]. Females of most NPFW species exit syconia through the hole that has been chewed by males of the pollinator species [31].

NPFWs are believed to require the presence of the pollinators for two reasons: (1) most fig species either abort or invest minimal resources into maturation of unpollinated or minimally pollinated syconia [12], and (2) most NPFW species lack morphological traits that would permit them to chew escape holes [32]. These observations imply that NPFWs that manage to find the correct species of fig tree, but in the absence of that fig’s pollinators, are doomed to die. Hence, it can be hypothesized that without specific traits that might allow persistence without the pollinator, specifically, mechanisms to prevent abscission of unpollinated syconia and mechanisms to escape once mature, NPFWs will not be encountered on figs outside their range that do not also have a persistent pollinator population. The reliance of most NPFW species on pollinators also dictates the relative abundance of fig wasp species within native ranges; pollinators tend to persist at a higher relative abundance than NPFWs [33,34,35], but there are exceptions due to abiotic conditions [36,37] and at range edges [38,39]. Fig wasp communities made up of exclusively NPFWs have been documented but are uncommon [23,40].

**Figure 1:**
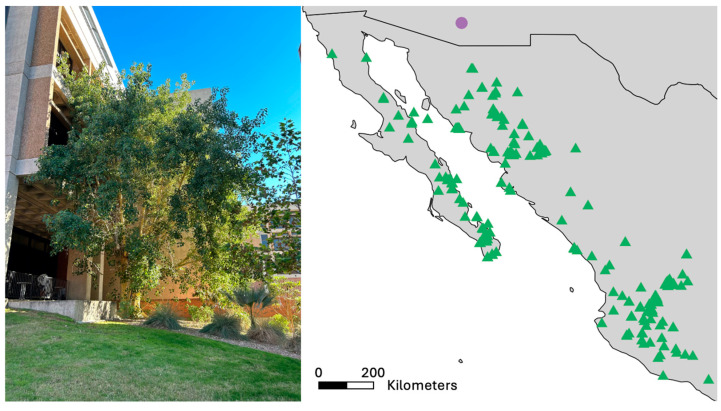
The *Ficus petiolaris palmeri* tree on University of Arizona campus (Tucson, AZ, USA), around 200 km from the known nearest conspecific in the native range and 150 km beyond the northern limit of its native range. Purple circle denotes location of the Tucson tree. Occurrence data (green triangles) from Botanical Information and Ecology Network (BIEN) v1.2.7 database using the RBIEN package [44]. Map generated using maps v3.4.3 [45] and ggplot2 v4.0.0 [46] packages in R v4.5.1 (13 June 2025) [47]. Photo by M. Gans.

On the University of Arizona campus in Tucson, Arizona, USA, a single *Ficus petiolaris* tree cultured from a seedling collected in Mexico was planted in the early 1980s, ca. 40 years ago [41]. Despite being ca. 200 km from the nearest known individual in its native range (Figure 1), fig wasps have been casually observed at this tree periodically for at least thirty years ([42]; J.L. Bronstein, pers. obs.). We have not been able to locate any other reproductive-age *F. petiolaris* individuals between the northern edge of the native range and Tucson, either by using online citizen scientist databases such as iNaturalist or by consulting local botanists, horticulturists, and plant enthusiasts.

We see two possible explanations for the apparently consistent presence of fig wasps on this fig tree. First, wasp individuals of one or more species may be continuously arriving from the native range, giving the appearance of persistence on this isolated tree while not in fact forming a persistent population (or populations). As highlighted above, pollinating fig wasps are well-documented to be blown exceptionally long distances; in contrast, very little is known about the dynamics of range extension by NPFWs (but see [23]). Second, if this fig tree is reproductively asynchronous in one critical way—bearing syconia that are releasing mature females at the same moment when other syconia are receptive to oviposition—then it is conceivable that pollinator wasps (and possibly NPFWs) could persist indefinitely in the absence of any other tree. To investigate these possibilities, we collected and dissected syconia monthly for 20 months to identify all wasp taxa present (both the unnamed pollinator [*Pegoscapus* sp.] and any NPFWs) and the reproductive asynchrony of the tree. We also utilized previously published mitochondrial DNA data for currently unnamed NPFW species in the species groups *Idarnes flavicolis* and *Idarnes carme* associated with *F. petiolaris* in its native range and collected from our focal Tucson tree to build a phylogenetic tree for *Idarnes*, including nine samples from the Tucson tree [42], 95 samples from the native range [43], and two samples in the same subfamily (Sycophaginae) as outgroups. We expected that if fig wasps did not form persistent populations in Tucson, then (a) there would be months in which no wasps would be present in any collected syconia, and (b) individuals of the NPFW species sampled from Tucson would show no evidence of genetic differentiation from the same wasp species in the native range. Additionally, we expected that if pollinating fig wasps do form persistent populations on this tree, the tree would be found to exhibit sufficient reproductive asynchrony to allow pollinator wasps to disperse from syconia and find receptive syconia within a few days. We expected that if NPFW wasps form persistent populations on this tree, then, in addition to the condition of within-tree reproductive asynchrony, the pollinator species, which prevents the abscission of unpollinated and insufficiently pollinated figs and facilitates NPFW dispersal from figs by chewing exit holes, would be present as well.

## 2. Results

### 2.1. Identity and Persistence of Wasp Community

We collected 446 syconia over the 20 months of the study (Table S1). Half showed signs of wasp larval development (50% C phase; phases described in Methods), contained mature wasps that had yet to emerge (26% D phase), and/or had exit holes and casings of wasp galls, indicating that wasps had developed within (13% E phase) (Table S1). We found foundresses, i.e., pollinator females (*Pegoscapus* sp.) that had oviposited and then died trapped inside, in only six syconia (1%) in three months (15% of the study; November 2023, December 2023, February 2024) (Table S1). No *Pegoscapus* sp. males were found in any syconium with mature wasps. Indeed, no syconium we sampled showed evidence that pollinator offspring of either sex had developed within. Further, no fig showed evidence of seed development (Table S1). Yet, we found wasp exit holes—widely assumed to be made by *Pegoscapus* males—in 64 syconia (14%) across all months except September 2023, October 2023, July 2024, and August 2024 (Table S1).

In contrast to the rarity of pollinators, the NPFW *Idarnes* spp. were exceedingly abundant. We found mature, ready-to-emerge or deceased *Idarnes* females in 177 syconia (40%) in every month we sampled (Table S1), with many of these syconia containing dozens of individual wasps. We found *Idarnes* males in all syconia with ready-to-emerge wasps. At no point in the study did we encounter any other NPFW taxa, although we did observe an unidentified Pteromalinae male outside of the study period.

Among syconia that had signs of *Idarnes* maturation, 24% exhibited characteristic wasp exit holes (Figure S1; Table S1). A small percentage of syconia we collected from the tree had external damage (<1%) that might have permitted wasp escape as well (Figure S1; Table S1), but we observed a much higher incidence (~13%) of external damage in figs collected from the ground (M. Gans, unpublished data). As a whole, these data suggest an absence of pollinators, an abundance of the NPFW *Idarnes*, and the presence of exit holes that are not constructed by males of the pollinator species.

### 2.2. Fig Phenology and Reproductive Asynchrony

We found two or more reproductive phases at every sampling date, with asynchrony ranging from a minimum of 1.29 in March 2024 to a maximum of 3.05 in December 2023 (any value larger than one indicates asynchrony, see Appendix A). However, we did not detect overlap between the two brief phases in which wasps emerge (phase D) and syconia are receptive (phase B) at any sampling date in the study. In fact, we did not find any syconia in the entire study period that could be definitively categorized as receptive, since this determination requires the presence of a live pollinator inside a syconium (see Methods). These data suggest that within-tree phenology is highly asynchronous, but it cannot be determined whether the tree is asynchronous in the way necessary for pollinators to persist on this single tree.

### 2.3. Idarnes Dispersal from the Native Range

**Figure 2:**
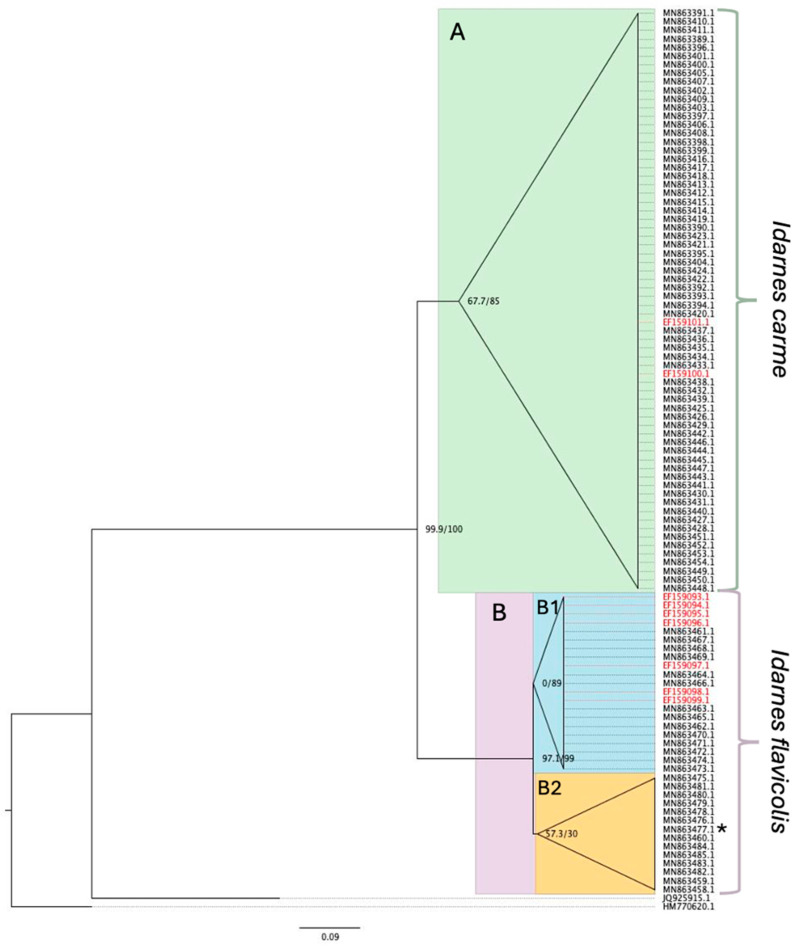
Maximum likelihood tree of *Idarnes* spp. associated with *Ficus petiolaris*. Taxa are identified by NCBI GenBank accession numbers. Taxa highlighted in red are samples from Tucson, Arizona. Clade A (green) are the *I. carme*-group species and clade B (purple) is the *I. flavicolis*-group species. Subclade B1 (blue) represents samples from Sonora, while subclade B2 (yellow) represents samples from Baja California, except for one sample from Sonora in B2 denoted by asterisk. Outgroups are *Anidarnes* sp. (JQ925915.1) and *Conidarnes* sp. (HM770620.1). Nodes are labeled with SH-aLRT and ultrafast bootstrap values, with relationships within clades collapsed for visual clarity.

Unnamed species from both the *Idarnes flavicolis* and *I. carme* species groups are present on the isolated Tucson tree (Figure 2). The *Idarnes* species found on the Tucson tree were genetically identical to the unnamed species known to associate with *F. petiolaris* in its native range [43]. We recovered two well-supported clades of the *I. flavicolis* species group and the *I. carme* species group in *F. petiolaris* (Clade B and A respectively, Figure 2), and two well-supported clades of the *I. flavicolis* species group originating from Baja California, Mexico and Sonora, Mexico (Clade B2 and B1 respectively, Figure 2). The delineation between the *I. carme* species group and the *I. flavicolis* species group, as well as the geographically delineated subclades of the *I. flavicolis* species group, are consistent with previous phylogeographic results using some of these data [43].

## 3. Discussion

Geographic ranges delineate the distributions of species and the interactions in which they participate. This raises an interesting question of when and how species engaged in obligate interspecific interactions can expand or shift their geographic ranges. One way that a species with an obligate partner species may persist beyond an apparent range boundary is by dispersing in parallel, either simultaneously or sequentially, with their obligate partner, resulting in the maintenance or reestablishment of the original interaction.

In this study, we sought to understand whether the obligate pollinator or any of its species-specific exploiter wasp species could persist on a single *Ficus petiolaris* located far north of its native range. Although there are additional *Ficus petiolaris* individuals in Tucson, Arizona, at nurseries, local museums, and botanic gardens, the single *Ficus petiolaris* on the University of Arizona campus is the only mature tree we have been able to locate. Wasp persistence on the Tucson tree could be explained either by the continuous dispersal of fig wasps from the native range, giving the illusion of persistent fig wasp populations on this isolated tree, or by within-tree reproductive asynchrony sufficient to allow wasps to persist by moving among syconia from generation to generation. Our results suggest that there is indeed a persistent fig wasp community on this tree. However, this community does not include the pollinator: rather, it is dominated by at least two species of non-pollinating (NPFW) *Idarnes* wasps, contrary to our prediction that pollinating fig wasps would have to be present for NPFWs to persist. Below, we first discuss how our results relate to our major question of persistence beyond the native range before discussing our surprising discovery of *Idarnes*’ ability to escape figs lacking pollinators. We conclude with the lessons our study offers for obligate interactions in a changing world.

We had expected that if fig wasps were continually dispersing from the native range (and did not form persistent populations in Tucson), there would be periods during our study when wasps were absent from the syconia we collected. *Pegoscapus* sp. was present briefly and intermittently throughout the study, consistent with periodic dispersal from the native range followed by local extinction. Conversely, one genus of NPFW, *Idarnes*, was present for the duration of our study (20 months). This would only be consistent with repeated dispersal from the native range if dispersal events were very common. In this case, the prevalence of *Idarnes* may be the result of greater dispersal capability than *Pegoscapus* sp. or the other NPFWs known to associate with *F. petiolaris* in its native range. *Idarnes*’ relatively long lifespans (up to one week) and ability to feed as adults on sugar water indoors may indicate a greater dispersal capacity than *Pegoscapus* [48]. Fig wasps are known to vary in their dispersal ability in relation to traits such as body size and fecundity [49]. While the dispersal capacities of *Idarnes* and *Pegoscapus* have not to our knowledge been directly compared, a greater dispersal capacity of *Idarnes* relative to *Pegoscapus* would corroborate our results.

We also had expected that continuous dispersal from the native range would result in individuals at the isolated tree that were more or as closely related to individuals in the native range than to each other. We did not see evidence of significant differentiation between *Idarnes* spp. individuals from Tucson and any part of the native range (Figure 2). Given the relatively young age of the tree in Tucson (planted ~40 years ago as a sapling) and the fact we only inferred relatedness using one gene, a lack of differentiation might have been expected. Other studies that have used phylogenetic data to explore fig wasp speciation and population structure have found mixed evidence for genetic isolation by distance or geographic barriers [20,50,51,52,53], probably due to their long dispersal distances [20,54]. Instead, differentiation in resource use, life history, and other adaptations may play larger roles in genetic differentiation among fig wasp populations [53,55]. Our results suggest that repeated dispersal is likely contributing to the presence of fig wasps in Arizona, particularly pollinating wasps, which were exceedingly rare across our study. Further, our preliminary work using genetic data on the origin of the *Idarnes* spp. in Tucson suggests that *I. flavicolis*-group wasps disperse from Sonora, Mexico (Figure 2). As there are no geographically defined clades of *I. carme*-group species, we are not able to definitively determine the location of origin of the *I. carme*-group individuals sampled. Dispersal from Sonora is not entirely surprising, as the closest known *F. petiolaris* conspecifics in the native range are in this region (Figure 1) and the prevailing winds at the study site in southern Arizona blow from the southeast year-round [56].

Consistent with the rarity of pollinating wasps, we found no evidence that the necessary condition for pollinator persistence on a single tree was met, namely, within-tree reproductive asynchrony allowing overlap between the developmental phases involving wasp dispersal from syconia (D) and pollinator wasp oviposition (B). We observed widespread within-tree asynchrony, similar to what has been observed previously in *F. petiolaris* and other *Ficus* species, including trees in populations at range edges [57,58,59,60]. The isolated Tucson tree is indeed highly asynchronous, with at least two developmental phases of syconia present at every sampling date, but at no point did we observe syconia in phase B and phase D on the same sampling date. However, it is important to note that syconium reproductive phases, particularly the classification of B phase, are defined by the presence of pollinators (see Methods), which we found to be exceedingly rare. Thus, it is possible that there is in fact sufficient within-tree reproductive asynchrony for pollinators to cycle on the tree, yet pollinator wasps do not form persistent populations on it for a different reason (e.g., inability to tolerate climatic conditions so far north of the native range). It is less clear what phenological conditions would permit persistence of NPFWs on a single tree. Some NPFWs have traits that could enable persistence with a broader range of reproductive asynchrony than pollinators; for example, whereas pollinator adults do not feed, *Idarnes* spp. females are known to survive for up to a week indoors on sugar water [48]. Further, their window of time for oviposition is considerably longer than the pollinators: they are known to oviposit before, during or even after the brief window of pollinator receptivity defined as B phase [61]. Longer lifespans and oviposition times could facilitate within-tree cycling for some species at the levels of within-tree phenological asynchrony we observed.

In addition to the condition of sufficient within-tree reproductive asynchrony, we predicted that NPFWs would only be able to persist when in the presence of the pollinating species, as pollination is believed to prevent abscission of syconia and exit holes chewed by males of the pollinator species allow adult NPFWs to disperse from mature figs. Surprisingly, we found evidence that at least one genus of NPFW (*Idarnes*) does not rely on pollinator species to perform either of these functions. Despite the rarity of the pollinating wasps (only six individuals in 20 months), *Idarnes* were extremely abundant throughout the study period; we found adult female *Idarnes* spp. in 40% of syconia, each containing up to dozens of individual wasps, in every month (Table S1). Furthermore, some of the syconia containing exclusively mature *Idarnes* males and females had exit holes, suggesting that not only pollinator males can chew exit holes in this system. Hole-chewing behavior has not to our knowledge been described previously in any *Idarnes* species. Taken together, these findings indicate that among the *Idarnes* species in this system are wasps capable of preventing abscission, chewing holes, or both.

There are a few intriguing possibilities for how these two functions, preventing syconium abscission and chewing exit holes, are performed by the *Idarnes* species in *F. petiolaris*. An experimental study in a different fig species (*Ficus citrifolia*) found that ~40% of figs exposed to only *I. flavicolis*-group wasps were not abscised and produced fully developed wasps, but *I. flavicolis*-group wasps were unable to naturally emerge from figs by chewing an exit hole [61]. In contrast, all *F. citrifolia* syconia exposed to only *I. carme*-group wasps were abscised before wasps fully developed [61]. Since syconia with only *I. carme*-group wasps were abscised, the ability of *I. carme*-group wasps to chew exit holes in the absence of pollinators could not be assessed. If these findings are relevant to *F. petiolaris* as well, this would suggest that the syconia we found containing only fully developed *Idarnes* wasps contained *I. flavicolis*-group wasps that prevented abscission. As for chewing exit holes, the *I. flavicolis*-group species present in *F. petiolaris* may have the capacity to chew holes, in contrast to the *I. flavicolis*-group species in *F. citrifolia*. Alternatively, it may be that *I. carme*-group wasps cannot prevent abscission, as is the case in *F. citrifolia*, but can chew their own exit holes. This second possibility is particularly intriguing because it would mean that neither *I. flavicolis*-group nor *I. carme*-group species can persist on their own in the absence of the pollinator, but could persist jointly without the pollinator. An experiment where syconia are exposed to both *I. carme*-group and *I. flavicolis*-group wasps would test this possibility.

While rarer than wasp exit holes, external holes created by birds may also contribute to the escape of *Idarnes* spp. (Figure S1). Over the course of the study, we observed house sparrows (*Passer domesticus*) pecking into syconia in the tree and on the ground. When we simulated the external damage created by birds using a pencil on developing figs that lacked wasp exit holes, *Idarnes* spp. females emerged from these figs (M. Gans, unpublished data). How commonly birds provide dispersal opportunities in natural settings is unclear, but warrants further investigation.

While our study documents the presence of *Idarnes* spp. without pollinators only on a single isolated fig tree, our work adds to an interesting pattern of antagonists preceding mutualists in establishment on isolated fig trees. Although this phenomenon has been minimally investigated, various NPFW species are known to persist in the absence of pollinators at several locations in the introduced range of *Ficus microcarpa* [23,38,40,62]. There is some evidence that this pattern may become magnified under climate change: the competitive interactions between the pollinating and non-pollinating fig wasps associated with *Ficus racemosa* altered in favor of NPFWs under simulated shifts in seasonal climate patterns, resulting in non-pollinator-dominated wasp communities [37].

Beyond the fig-fig wasp mutualism, our results provide insight into the factors that determine which interactions persist outside of native ranges. As in many systems in which dispersal distances are large (e.g., marine broadcast spawners and wind-dispersed plants), fig wasp colonization of a new habitat likely depends more on the suitability of conditions than dispersal opportunity. While much work focuses on how thermal tolerance will affect species’ ability to respond to climate change, other aspects of an organism’s life history, such as its dispersal strategies and lifespan, may also play important roles in determining a species persistence beyond current ranges. Despite growing evidence in diverse systems that differences in thermal tolerance between species have the capacity to disrupt species interactions under climate change scenarios [7,8,63], little is known about the kinds of species interactions that are most susceptible to breaking down due to thermal mismatch [64]. We encourage work across systems to investigate whether properties of species interactions (net effect, dependence, extent of physiological integration, etc.) influence the ability of species and interactions to respond to climate change through range shifts.

## 4. Materials and Methods

### 4.1. Study System

**Figure 3:**
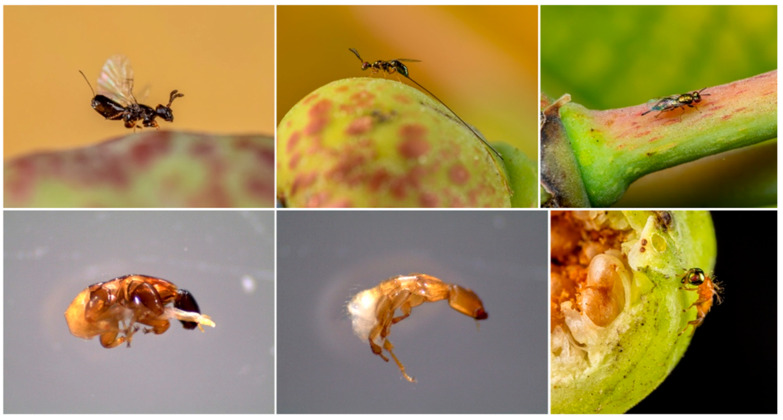
Common wasp genera associated with *F. petiolaris*. Top row, from left to right: females of *Pegoscapus* (pollinator), *Idarnes* (non-pollinator), and an unidentified Pteromalinae (likely *Heterandrium*) (non-pollinator). Bottom row: males of *Pegoscapus* sp., *Idarnes* sp., and an unidentified Pteromalinae (wingless morph). All wasp bodies (excluding ovipositors) are less than 3 mm in length. Photos of *Pegoscapus* and *Idarnes* males by Justin van Goor, all other photos by Bruce Taubert.

*Ficus petiolaris* Kunth (Moraceae, subgenus *Urostigma*, section *Americana*), commonly known as the Sonoran rock fig or Baja rock fig, is endemic to mainland Mexico and the Baja Peninsula (Figure 1). There are currently two recognized subspecies, *F. petiolaris palmeri* and *F. petiolaris petiolaris* [65]. *F. petiolaris* is pollinated by a single unnamed species of *Pegoscapus* (superfamily Chalcidoidea, family Agaonidae). At least nine species of NPFW from three families are associated with *F. petiolaris* (Figure 3) [43]. *Idarnes* (Sycophaginae, Pteromalidae) are characterized by the females’ long ovipositors, which they use to lay eggs through the wall of the syconium. Although the *Idarnes* species associated with *F. petiolaris* are currently unnamed, there are thought to be four species from two species groups, *Idarnes flavicolis* and *I. carme*. These species groups differ in resource use: *I. flavicolis*-group species are gallers active at the time of pollination and *I. carme*-group species are kleptoparasites that oviposit after pollination and feed on the gall tissue induced by pollinators [61,66]. *F. petiolaris* also hosts two unnamed species of *Heterandrium* (Pteromalinae, Pteromalidae), an unnamed species of *Ficicola* (Pteromalinae, Pteromalidae), an unnamed wasp parasitoid in the genus *Physothorax* (Torymidae), and an unnamed species of *Sycophila* (Eurytomidae) [43].

### 4.2. Fig Phenology and Fig Wasp Dynamics

We tagged 19 branches throughout the lower canopy of the focal tree, of which 14 produced syconia throughout the study (20 months). Each month, we selected up to two of the most mature syconia from each tagged branch, as these are more likely to have wasps developing within. When branches had few, very small, or no syconia, we selected either one syconium or none. Because we preferentially sampled larger, older syconia, our sampling likely underestimates the proportion of syconia in early phases of development. Collected syconia were inspected for external damage and wasp exit holes before halved and examined under a dissecting microscope. Phases were denoted by the standard A–E scheme used by fig biologists (e.g., [67]) (Table 1). Pollinators can persist on their natal tree (i.e., maintain a reproducing population across generations) only if phases B and D coincide. We noted the presence or absence of a pollinator (foundress) body, as well as the genus of mature wasps present when possible (late C, D, and sometimes E phase figs). Syconia containing live adults (usually D- or E-phase figs) were placed in glass vials, covered with mesh bags, and left for 24 h to allow emergence before adding 70% ethanol to the vial for storage. At the end of the study, we inspected vial contents under a dissecting microscope to confirm wasp identifications. Although previous studies have used morphology to distinguish among *Idarnes* species groups, we did not do so, as these identifications can be unreliable (Carlos Machado, pers. comm.).

### 4.3. Fig Wasp Biogeography

To determine the origin of the *Idarnes* taxa found on the focal tree, we built a phylogenetic tree using previously published genetic sequences from *Idarnes* samples associated with *F. petiolaris*, including nine samples from the tree in Arizona previously unincluded in phylogenies of *Idarnes* samples associated with *F. petiolaris*. We focused on *Idarnes* because of their high frequency at the focal tree (see Results) and the large number of sequences available online; *Idarnes* species are associated with most or all neotropical *Ficus* species and have been exceptionally well-studied [e.g., [69]]. Sequences of *Idarnes* samples associated with *F. petiolaris* included in the phylogenetic tree were downloaded from NCBI GenBank (MN863389–MN863485; EF159093–EF159101) [42,43]. We also downloaded two sequences from NCBI GenBank to include as outgroups: *Conidarnes* sp. (Sycophaginae, Pteromalidae) (HM770620) and *Anidarnes* sp. (Sycophaginae, Pteromalidae) (JQ925915). No new sequences were produced as part of this study. We aligned sequences using MAFFT v7.471 with the maximum number of iterative refinement at 1000 [70] and trimmed the alignment using trimAl v1.5.rev0 using -gt 0.5 to specify that sites with less than 50% sequences present should be removed [71]. We visually inspected the untrimmed and trimmed alignments using seaview v5.1 to ensure quality of alignment [72]. To build the phylogenetic tree, we used IQ-TREE 2 v2.1.2, assigning branch supports using SH-aLRT test and ultrafast bootstrap with 1000 replicates [73]. The phylogenetic tree was rooted and visualized using FigTree v1.4.4 http://tree.bio.ed.ac.uk/software/figtree/ (accessed on 5 December 2025).

## Figures and Tables

**Table 1:** Developmental phases used to classify syconia in this study, based on original definitions by Galil and Eisikowitch 1968 [68].

Phase Name	Description
A	Pre-reproductive phase, no sign of wasp activity (ovules are small, stigma surfaces are white)
B	Female reproductive phase, pollinator wasp inside fig actively pollinating or ovipositing
C	Developmental phase, ovules are enlarged and discolored with wasp larvae or seeds developing inside
D	Male reproductive phase, fully developed wasps emerging inside the syconium and dispersing if exit hole present
E	Post-reproductive, fig is ripe and inside are seeds or empty ovules where wasps emerged from

## Data Availability

The original contributions presented in this study are included in the article/Supplementary Materials. Further inquiries can be directed to the corresponding author.

## Supplementary Materials

The following supporting information can be downloaded at: https://www.mdpi.com/article/10.3390/plants15071012/s1, Table S1: Monthly data from collected syconia at each sampling date throughout the study; Figure S1: Contrasting wasp exit holes (left) and large, external holes made by birds (right) observed in our study.

## Abbreviations

The following abbreviations are used in this manuscript:

NPFW	Non-pollinating fig wasp

## Appendix A

Using our phenological data, we measured within-tree reproductive asynchrony for each sample date using an evenness index (the inverse Simpson’s diversity) from [45]:

(A1) $$\mathrm{Evenness}\;=\;\frac{1}{p_{A}^{2}+p_{B}^{2}+p_{C}^{2}+p_{D}^{2}+p_{E}^{2}}$$

Here, $p_{i}$ is the proportion of syconia in phase *i*. Any value over one is considered asynchronous, with the number of phases (5) equal to the maximum asynchrony value.
