# Supplementary material for: Range Expansion of Obligate Antagonists but Not Mutualists in a Desert Fig (Ficus petiolaris)
Source: Plants (Basel). 2026 Mar 26;15(7):1012. doi: 10.3390/plants15071012 (PMC13074616; doi:10.3390/plants15071012)
Supplement: Supplementary file 1 [file plants-15-01012-s001.zip › plants-4121734-supplementary.pdf]

Table S1: Monthly data from collected syconia at each sampling date throughout the study. Each cell denotes the number of syconia collected on that data in a particular phenological phase (A-E) or the number of syconia with mature female wasps, exit holes, seeds, or external damage present. Asynchrony index for each month was calculated using the method described in Appendix 1.

**Date**	**A phase**	**B phase**	**C phase**	**D phase**	**E phase**	***Pegoscapus* sp.**	***Idarnes* spp.**	**Exit holes**	**Seeds**	**External damage**	**Asynchrony index**	**Sample size**
9/21/23	10	0	18	3	0	0	3	0	0	0	2.23	31
10/19/23	2	0	8	17	0	0	21	0	0	0	2.03	27
11/9/23	0	0	8	17	3	2	17	3	0	0	2.14	28
12/14/23	1	0	11	6	9	2	6	13	0	0	3.06	27
1/19/24	1	0	13	5	4	0	5	3	0	0	2.48	23
2/16/24	0	0	10	6	1	2	8	1	0	0	2.11	17
3/25/24	0	0	14	1	1	0	1	1	0	0	1.28	16
4/30/24	12	0	6	0	9	0	1	9	0	0	2.85	27
5/17/24	1	0	10	1	1	0	3	1	0	0	1.63	13
6/12/24	1	0	22	3	0	0	4	1	0	0	1.35	26
7/12/24	9	0	11	0	0	0	4	0	0	0	1.98	20
8/13/24	12	0	10	0	0	0	3	0	0	0	1.98	22
9/19/24	0	0	11	8	3	0	12	3	0	0	2.51	22
10/16/24	0	0	13	5	6	0	9	6	0	2	2.51	24
11/14/24	0	0	18	5	1	0	22	1	0	1	1.64	24
12/12/24	3	0	3	18	0	0	19	1	0	0	1.69	24
1/16/25	0	0	12	9	0	0	10	2	0	0	1.96	21
2/15/25	0	0	11	3	8	0	10	8	0	0	2.51	22
3/20/25	0	0	12	2	2	0	7	1	0	0	1.69	16
4/22/25	0	0	1	6	9	0	12	10	0	1	2.17	16
**Total**	52	0	222	115	57	6	177	64	0	4	–	446

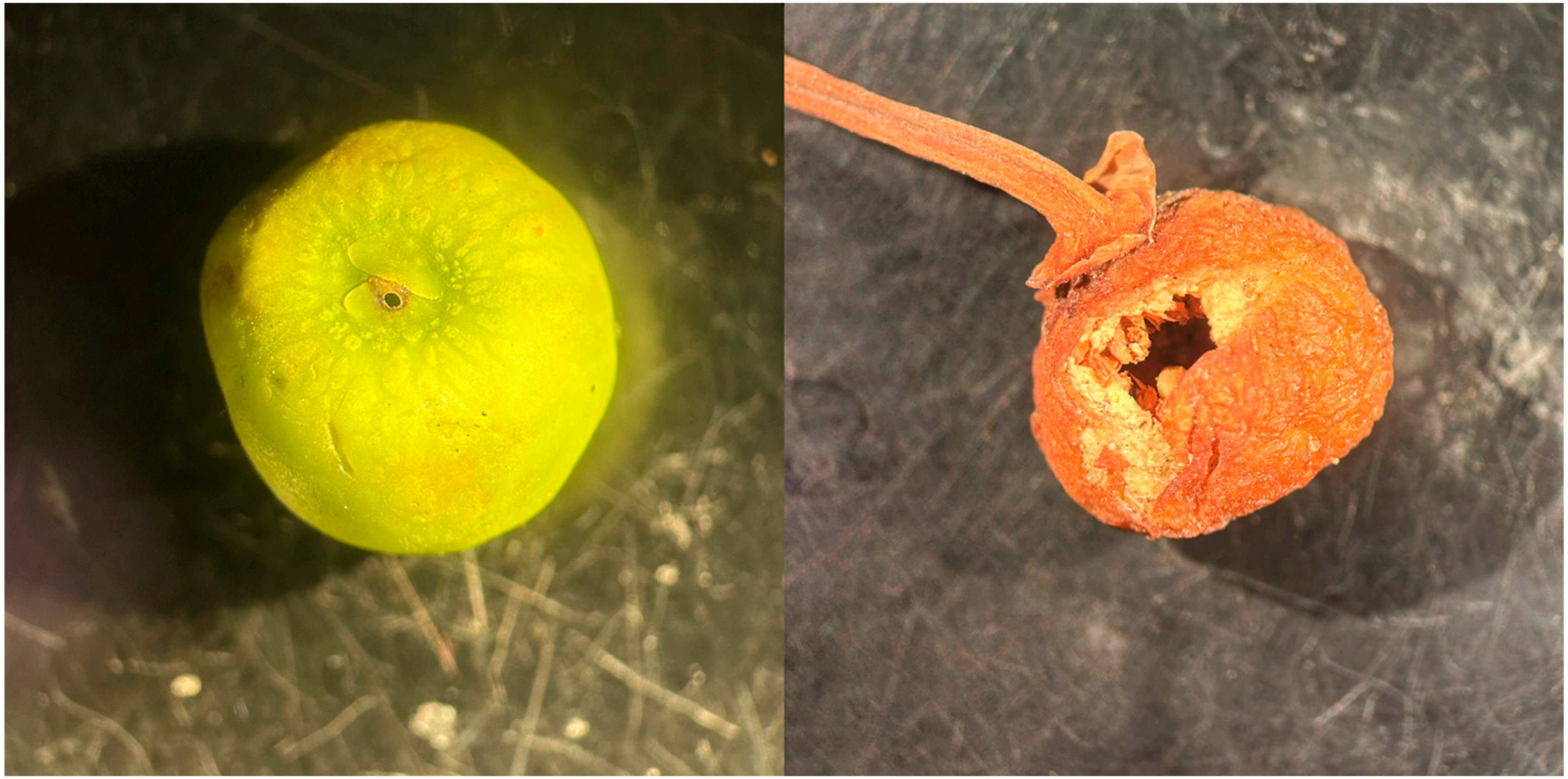

Figure S1. Contrasting wasp exit holes (left) and large, external holes made by birds (right) observed in our study. Photos by M. Gans and J. Bronstein, respectively.
